# Effects of Low-Flow Sevoflurane Anesthesia on Pulmonary Functions in Patients Undergoing Laparoscopic Abdominal Surgery

**DOI:** 10.1155/2016/3068467

**Published:** 2016-06-20

**Authors:** Cihan Doger, Kadriye Kahveci, Dilsen Ornek, Abdulkadir But, Mustafa Aksoy, Derya Gokcinar, Didem Katar

**Affiliations:** ^1^Department of Anesthesiology and Reanimation, Ankara Ataturk Training and Research Hospital, 06800 Ankara, Turkey; ^2^Department of Anesthesiology and Reanimation, Ulus State Hospital, 06050 Ankara, Turkey; ^3^Department of Anesthesiology and Reanimation, Ankara Numune Training and Research Hospital, 06230 Ankara, Turkey; ^4^Department of Anesthesiology and Reanimation, Faculty of Medicine, Yildirim Beyazit University, 06800 Ankara, Turkey; ^5^Department of Pulmonology, Ankara Yenimahalle State Hospital, 06370 Ankara, Turkey

## Abstract

*Objective*. The aim of this prospective, randomized study was to investigate the effects of low-flow sevoflurane anesthesia on the pulmonary functions in patients undergoing laparoscopic cholecystectomy.* Methods*. Sixty American Society of Anesthesiologists (ASA) physical status classes I and II patients scheduled for elective laparoscopic cholecystectomy were included in the study. Patients were randomly allocated to two study groups: high-flow sevoflurane anesthesia group (Group H, *n* = 30) and low-flow sevoflurane anesthesia group (Group L, *n* = 30). The fresh gas flow rate was of 4 L/min in high-flow sevoflurane anesthesia group and 1 L/min in low-flow sevoflurane anesthesia group. Heart rate (HR), mean arterial blood pressure (MABP), peripheral oxygen saturation (SpO_2_), and end-tidal carbon dioxide concentration (ETCO_2_) were recorded. Pulmonary function tests were performed before and 2, 8, and 24 hours after surgery.* Results*. There was no significant difference between the two groups in terms of HR, MABP, SpO_2_, and ETCO_2_. Pulmonary function test results were similar in both groups at all measurement times.* Conclusions*. The effects of low-flow sevoflurane anesthesia on pulmonary functions are comparable to high-flow sevoflurane anesthesia in patients undergoing laparoscopic cholecystectomy.

## 1. Introduction

The use of the low-flow anesthesia technique provides protection of the heat and humidity of the respiratory system, while minimizing cost and preventing air pollution. The physiology of the tracheobronchial environment is protected as mucociliary clearance is more excellently maintained in a low-flow anesthesia technique than in high-flow anesthesia [1–4]. Moreover, as the waste gases are reduced, atmospheric pollution is lowered, health risks for operating room staff are decreased, and ecological balances are maintained. However, possible disadvantages resulting from the inappropriate use of low-flow anesthesia include hypoxia, excessive or insufficient concentrations of volatile agents, hypercapnia, and the accumulation of potentially toxic gases. Low-flow anesthesia is the issue before the Y piece as connection point with tube/circle system. That means that if low-flow anesthesia is used successfully in a patient, it will never have different effects on pulmonary functions in comparison with higher flow anesthesia. It only affects the waste gas amount normally.

Laparoscopic techniques compared with open surgery are more widely preferred in the surgical field, because they require a shorter hospital stay and cause less morbidity and mortality [5]. In laparoscopic surgery, the field of interest may be visualized by forming an artificial pneumoperitoneum. The pneumoperitoneum is created by insufflating CO_2_ into the peritoneal cavity at a mean flow rate of 1-2 L/min. The pneumoperitoneum has both mechanical and chemical effects on the pulmonary system. With the first insufflation, intra-abdominal pressure is increased, causing the diaphragm to be distended upward. This distension increases intrathoracic pressure, and the alveoli are collapsed. During the laparoscopy, the direct effects of the additional positive end-expiratory pressure cause a hemodynamic imbalance [5–7].

Few studies have explored the low-flow sevoflurane anesthesia technique for laparoscopic abdominal surgery. In this study, we aimed to investigate the effects of low-flow sevoflurane anesthesia on pulmonary functions in patients who underwent laparoscopic cholecystectomy.

## 2. Methods

Approval for this study was obtained from the Ethics Committee at the University of Yildirim Beyazit, Faculty of Medicine, with number 2012-T4137-03-333. It conforms to the provisions of the Declaration of Helsinki. Written informed consent was obtained from all patients. Seventy patients (20–55 years old) scheduled to have elective laparoscopic cholecystectomy and classified as American Society of Anesthesiologists physical status class I or II were prospectively enrolled. Patients with obstructive pulmonary disease and cardiac or renal or hepatic disease, smokers, and those fasting for more than 12 hours were excluded. A web application (https://www.randomizer.org/) was used for randomization. Patients were randomly allocated to two study groups: high-flow sevoflurane anesthesia group (Group H) and low-flow sevoflurane anesthesia group (Group L). The patients were blind to which group they were in, but the intraoperative anesthesia team were informed about anesthesia and method. But both postoperative pulmonology and anesthesiology doctor, who evaluated pulmonary function tests, were blinded.

### 2.1. Anesthesia

All patients were premedicated with midazolam at a dose of 0.02 mg/kg intravenously (IV) 30 minutes before the operation. Heart rate (HR), noninvasive mean arterial blood pressure (MABP), peripheral oxygen saturation (SpO_2_), and end-tidal carbon dioxide concentration (ETCO_2_) were monitored. All patients were induced by fentanyl (1 *µ*g/kg IV) and propofol (3–5 mg/kg IV) and muscle relaxation was achieved by administration of atracurium (0.5 mg/kg IV). Anesthesia was maintained at 3.4% sevoflurane and 50% nitrous oxide in oxygen. Patients in Group H received fresh gas flow of 4 L/min throughout the procedure. Patients in Group L received a fresh gas flow rate of 4 L min^−1^ for the first 10 min and then were maintained with a fresh gas flow of 1 L/min. All patients were mechanically ventilated with a tidal volume of 8 mL/kg and a frequency of 12 breaths/min with the ventilatory (Dräger® Julian, Lübeck, Germany) rate adjusted to maintain ETCO_2_ of 30 to 40 mmHg. Atracurium (10 mg IV) was administered every 20 minutes. Hemodynamic functions were maintained within 20% of baseline by adjusting supplemental fentanyl injection, as well as vasoactive drugs as needed. Sevoflurane was discontinued with the beginning of the skin sutures and the fresh gas flow was changed to 6 L/min of oxygen. Neuromuscular blockade was reversed by administering neostigmine (0.04 mg/kg IV) and atropine (0.02 mg/kg IV). The patient was extubated when the patient met the criteria for tracheal extubation (respiratory rate >8, spontaneous breathing with a minimum of 8 mL/kg body weight, ability to sustain a 5 s head lift, sustained hand grip, and sustained arm lift). Fresh barium hydroxide was used to fill the carbon dioxide-absorbent canister before each patient.

### 2.2. Measurements

Pulmonary function tests were performed before and after the surgery at 2nd, 8th, and 24th hours. Forced vital capacity (FVC, L), forced expiratory volume in 1 s (FEV1, L), and forced expiratory volume in 1 s/forced vital capacity (FEV1/FVC %) values were measured using the spirometer (KoKo Legend 314000 Spirometer, nSpire Health, USA) by the same physician and the patients performed the forced expiratory maneuver at least three times in the sitting position. The pulmonary function test results were expressed as percentages of the expected values adjusted for age, height, body weight, and sex [8]. HR, MABP, and SpO_2_ were monitored and recorded after tracheal intubation and at 5 min intervals during intraoperative period. ETCO_2_ values were noted 5 min (T1), 10 min (T2), 20 min (T3), 30 min (T4), 40 min (T5), 50 min (T6), and 60 min (T7) after tracheal intubation. The anesthetist who evaluated and recorded the data was blind to the group allocation.

### 2.3. Statistical Analysis

The power value was evaluated with the G-power 3.1 package program. An a priori power analysis was based on previously published study [8]. The sample sizes were calculated on the assumption that a 20% difference in FEV1 and FVC was significant. In accordance with the power calculation method, to demonstrate a 20% difference at *α* = 0.05 and a power of 85%, 30 patients per group were required. Statistical evaluation of the data was performed using SPSS ver. 16.0 (SPSS, Chicago, IL, USA). Kolmogorov-Smirnov test was used to identify the distribution of variables. Results are expressed as mean ± SD or ratio of patients. Comparison between the two groups was performed using Mann-Whitney *U*, Wilcoxon Sign Test, or paired *t*-test. The results were evaluated at a 95% confidence interval at a significance level of *P* < 0.05.

## 3. Results

Three patients with lung disease, one patient with cardiac disease, three smokers, and one patient switching from laparoscopic cholecystectomy to open cholecystectomy were excluded. Also two of the patients, who had operations lasting longer than one hour because of surgical problems that may result in altering the postoperative pulmonary function tests, were excluded. Sixty patients completed the study. The demographic data are shown in Table 1. The groups were similar in demographic characteristics.

No significant differences were found between the two groups in terms of HR, MABP, SpO_2_, and ETCO_2_ during anesthesia. Although mean ETCO_2_ levels in Group L were higher than those in Group H, the difference was not statistically significant. Higher CO_2_ levels did not result in clinical complication in any patient.

Preoperative and postoperative pulmonary function test results were similar in both groups. In all patients, the FVC and FEV1 values were significantly decreased in all measurements in the postoperative period compared to preoperative values, and this decrease was most pronounced during the second hour of the postoperative period. We also compared the postoperative FEV1/FVC ratio to the preoperative values and found no statistically significant differences between the two groups (Table 2).

There was not any contamination whereby the protocols were altered for any patients in either the low-flow or the high-flow groups with regard to the intervention specifically during the procedure. None of the patients complained about any side effect of anesthesia or its complications.

## 4. Discussion

The present study shows that the effects of low-flow sevoflurane anesthesia on pulmonary functions in patients undergoing laparoscopic cholecystectomy were comparable to the effects of high-flow sevoflurane anesthesia. Laparoscopic cholecystectomy is often used as an alternative to laparotomy due to its advantages, including shorter hospital stays, earlier mobilization, smaller incisions, a low incidence of postoperative pain, and less need for analgesics [5, 6]. During laparoscopic cholecystectomy, as a result of CO_2_ insufflation, intra-abdominal pressure is increased, the movement of the diaphragm is restricted, and the functional residual capacity (FRC) is decreased. Hypercarbia can also occur due to decreased FRC and CO_2_ absorption [6, 7]. Baraka et al. [7] reported that, during the laparoscopic interventions, end-tidal CO_2_ showed a progressive increase following induction when the ventilation parameters were constant. Low-flow anesthesia has some disadvantages, such as hypoxia, overdose of anesthetic agent, and hypercapnia [9]. At the onset of low-flow anesthesia, the clearance of nitrogen from the blood by giving high-flow 100% O_2_ ventilation for 10–20 minutes ensures an easier distribution of the anesthetic gases into the system and a more convenient adjustment of the gas concentrations. It has been demonstrated that the replacement of nitrogen with oxygen in the lungs increased the oxygen reserve and thereby the apnea time [10]. In our patients, although ETCO_2_ levels during the low-flow anesthesia period were found to be higher in Group L than in Group H, the difference was not statistically significant, and increased CO_2_ levels did not result in clinical complications. With a decreasing flow rate, the difference between the concentration of O_2_ in the fresh gas mixture and the concentration of O_2_ in the inspired air is increased. If low FiO_2_ is inspired during rebreathing, a potential risk of hypoxia occurs. To definitively prevent hypoxemia and to ensure a continuous and adequate O_2_ presentation, the concentration of O_2_ in the inspirium should be at least 30% [10]. Okada et al. [11] in their low-flow anesthesia sevoflurane study found that the difference between FiO_2_ and O_2_ increased with time as the inspired oxygen concentration (FiO_2_) values increased. However, reductions in FiO_2_ to less than 30% were not seen in any patients in the two groups.

In our study, we used a mixture of 50% O_2_ and 50% N_2_O. The concentration of O_2_ in the inspired air was monitored. In none of the subjects did FiO_2_ fall below 42% during the operation. Although decreases in the concentration of inspired and expired O_2_ were observed, it never fell to a clinically significant level.

As inspired and expired gases are mixed during the administration of low-flow anesthesia, the concentration of inspired gas is not directly correlated with the settings of the vaporizer. One of the important dangers of the low-flow anesthesia technique is the elevation of circulating the anesthetic concentration to levels so much higher than the concentrations set in the vaporizer [12, 13]. However, during the administration of low-flow anesthesia, it is not very likely to reach a high concentration in a short period of time, even if the settings of the vaporizer are erroneous, because the concentration of the anesthetic agent is slowly increased in the continuously breathed air in the pulmonary tract. Unlike high-flow anesthesia, a careful clinical observation may serve to determine the erroneous settings before serious and probably dangerous changes have occurred.

During low-flow anesthesia, the ventilation pressure may change. Negative pressure may occur and tidal volume may be decreased. In the studies performed with low-flow anesthesia, ETCO_2_ was maintained between 30 and 35 mmHg. Sajedi et al. [14] did not find any difference in the SPO_2_ and ETCO_2_ values with low- and high-flow anesthesia in laparoscopic cholecystectomy patients. In our patients, ETCO_2_ varied between 26 and 40. As the change of ETCO_2_ did not cause a change in SPO_2_ or any hemodynamic parameters, we did not make any changes to the tidal volume and pulmonary rate settings.

In the techniques of low-flow anesthesia, vector gases and volatile anesthetics are known to provide considerable cost savings due to the decreased amount of wasted gas. In previous studies, it has been reported that 80–90% of anesthetic agents and gases are wasted with a flow rate of 5 L/min [15].

In low-flow anesthesia, it is easier to maintain the anesthetic depth previously obtained in volatile anaesthetics with low solubility, such as desflurane and sevoflurane. Furthermore, when high-flow is used, some issues may be encountered with the agents that react with dry CO_2_ absorbents, such as desflurane, enflurane, and sevoflurane [16].

The preservation of hemodynamic stability has been proven by some researchers in recent low-flow anesthesia studies [17–19]. In our study, hemodynamic stability was preserved and no significant difference in results was observed between the groups.

Physiological changes during general anesthesia could lead to postoperative pulmonary complications. A decrease in FRC leads to a disruption in the balance between ventilation/perfusion and hypoxemia. A decrease in postoperative values of VC and FRC has been shown in many high-flow anesthesia studies which investigated postoperative pulmonary function tests after abdominal surgery [20]. Rock and Rich [21] showed a decrease in FVC and FRC after upper abdominal and tracheal surgeries. In another study, preoperative and postoperative FVC, FEV1, and FEV1/FVC values were measured on the first and sixth day in open and laparoscopic cholecystectomy patients and it was shown that in open cholecystectomy patients FVC, FEV1, and FEV1/FVC values were lower than in the other groups [22].

A few groups have studied the effects of low-flow anesthesia on hemodynamic and pulmonary functions and in these studies isoflurane and desflurane were used as volatile anesthetic agents [18, 23]. Bilgi et al. [1] compared FVC and FEV1 levels after low-flow and high-flow anesthesia with desflurane as the inhalation anesthetic agent and observed a decrease in FVC and FEV1 levels in both groups on the postoperative first day, but, in the low-flow anesthesia group, the decrease was much greater than in the high-flow anesthesia group. In our study, although postoperative FVC and FEV1 levels at the 2nd, 8th, and 24th hours were lower than preoperative levels, there was no statistically significant difference between the two groups (*P* > 0.05). The decrease was markedly higher at the 2nd hour than at the 8th and 24th hours. The causes of a significant decrease at the 2nd postoperative hour may be due to a prolonged effect of myorelaxants or recurarisation, postoperative pain, or increased abdominal pressure due to intraoperative-caused pneumoperitoneum.

Our facility is not enough because the study has limitations. It would be useful if we had measured the gas level and the heat/humidity after the “Y” piece. But we could not do it. If high- or low-flow anesthesia is applied properly, no different gas composition and no pressure or volume changes after the “Y” piece are found. But when it is not used with care, it can affect both oxygenation and ventilation and can cause serious damage to the patient. When we use low-flow anesthesia, it can better preserve heat and humidity compared to high-flow. This difference can impact on pulmonary functions as well. But this kind of harming effect needs time to be meaningful. In this study, average operating time is approximately 47 min so that is enough to affect the pulmonary functions. Fortunately, we did not see the effect on pulmonary functions.

Consequently, neither the low-flow sevoflurane group nor the high-flow sevoflurane group showed any significant change in pulmonary functions postoperatively. In light of these findings, we conclude that low-flow sevoflurane anesthesia, without any adverse effect, may be administered for laparoscopic abdominal surgery.

## Figures and Tables

**Table 1:** Demographic characteristics and findings.

	Group H (*n* = 30)	Group L (*n* = 30)	*P* value
Age (years)	44.77 ± 9.21	44.57 ± 9.87	0.936
Sex (male/female)	3/27	3/27	1.000
Weight (kg)	76.4 ± 9.42	75.9 ± 11.98	0.858
Height (cm)	164.13 ± 7.08	163.10 ± 5.50	0.530
Body mass index	28.56 ± 4.5	28.67 ± 5.12	0.927
ASA class (I/II)	20/10	19/11	0.788
Operative time (min)	47.93 ± 4.91	46.28 ± 18.5	0.787

Values are expressed as ratio or mean ± SD.

Group H: high-flow sevoflurane anesthesia group.

Group L: low-flow sevoflurane anesthesia group.

**Table 2:** Comparison of the preoperative and postoperative forced expiratory volume in 1 s forced vital capacity and forced expiratory volume in 1 s/forced vital capacity values.

	Group H	Group L	*P* value
FEV1 (L)			
Preoperative	2.85 ± 0.69	2.78 ± 0.54	0.643
Postoperative 2 h	2.37 ± 0.73	2.22 ± 0.54	0.723
Postoperative 8 h	2.44 ± 0.68	2.46 ± 0.54	0.865
Postoperative 24 h	2.69 ± 0.78	2.72 ± 0.56	0.868

FVC (L)			
Preoperative	3.13 ± 0.74	3.10 ± 0.57	0.904
Postoperative 2 h	2.51 ± 0.84	2.47 ± 0.48	0.756
Postoperative 8 h	2.73 ± 0.77	2.69 ± 0.52	0.805
Postoperative 24 h	2.95 ± 0.79	2.96 ± 0.55	0.952

FEV1/FVC (%)			
Preoperative	0.92 ± 0.10	0.90 ± 0.07	0.272
Postoperative 2 h	0.93 ± 0.06	0.90 ± 0.08	0.074
Postoperative 8 h	0.91 ± 0.09	0.92 ± 0.05	0.473
Postoperative 24 h	0.91 ± 0.10	0.91 ± 0.07	0.988

Values are expressed as mean ± SD.

FEV1: forced expiratory volume in 1 s.

FVC: forced vital capacity.

FEV1/FVC: forced expiratory volume in 1 s/forced vital capacity.
